# Dopaminergic Modulation of the Voltage-Gated Sodium Current in the Cochlear Afferent Neurons of the Rat

**DOI:** 10.1371/journal.pone.0120808

**Published:** 2015-03-13

**Authors:** Catalina Valdés-Baizabal, Enrique Soto, Rosario Vega

**Affiliations:** Instituto de Fisiología, Benemérita Universidad Autónoma de Puebla, Puebla, México; University of North Dakota, UNITED STATES

## Abstract

The cochlear inner hair cells synapse onto type I afferent terminal dendrites, constituting the main afferent pathway for auditory information flow. This pathway receives central control input from the lateral olivocochlear efferent neurons that release various neurotransmitters, among which dopamine (DA) plays a salient role. DA receptors activation exert a protective role in the over activation of the afferent glutamatergic synapses, which occurs when an animal is exposed to intense sound stimuli or during hypoxic events. However, the mechanism of action of DA at the cellular level is still not completely understood. In this work, we studied the actions of DA and its receptor agonists and antagonists on the voltage-gated sodium current (I_Na_) in isolated cochlear afferent neurons of the rat to define the mechanisms of dopaminergic control of the afferent input in the cochlear pathway. Experiments were performed using the voltage and current clamp techniques in the whole-cell configuration in primary cultures of cochlear spiral ganglion neurons (SGNs). Recordings of the I_Na_ showed that DA receptor activation induced a significant inhibition of the peak current amplitude, leading to a significant decrease in cell excitability. Inhibition of the I_Na_ was produced by a phosphorylation of the sodium channels as shown by the use of phosphatase inhibitor that produced an inhibition analogous to that caused by DA receptor activation. Use of specific agonists and antagonists showed that inhibitory action of DA was mediated both by activation of D1- and D2-like DA receptors. The action of the D1- and D2-like receptors was shown to be mediated by a G_αs_/AC/cAMP/PKA and G_αq_/PLC/PKC pathways respectively. These results showed that DA receptor activation constitutes a significant modulatory input to SGNs, effectively modulating their excitability and information flow in the auditory pathway.

## Introduction

The organ of Corti consists of several cells types that perform auditory functions harmoniously. The hair cells are responsible for the sensory transduction and synaptic activation of the afferent neurons. The outer hair cells (OHC) play a role mainly related to the cochlear amplifier, while the inner hair cells (IHC) fundamentally function in the detection of auditory stimuli. The IHC receive afferent innervation from type I spiral ganglion neurons (SGNs), which comprise approximately 95% of the cochlear afferents [1], while the OHC receive afferent innervation through the type II SGNs, which correspond to approximately 5% of the cochlear afferents.

The efferent neurons originating from the lateral superior olivary complex (LOC) make synaptic contacts with the afferent neurons innervating the IHC [2]. These neurons have been found to release various neurotransmitters including acetylcholine, dynorphin [3], encephalin [4], calcitonin gene-related peptide (CGRP) [5], GABA [6], adenylate cyclase-activating polypeptide [7], and dopamine (DA) [8]. Another group of efferent neurons originates from the medial superior olivary complex (MOC) and synapse directly onto the OHC [9]. The MOC efferents release acetylcholine [3], GABA [6] and CGRP [10]. A particular case consists of a group of neurons originating from the periolivary nucleus, which form part of the efferent system innervating both the OHC and IHC that release serotonin as a neurotransmitter [11].

The type I SGNs express D1, D2, D4 and D5 dopaminergic receptor subtypes [12,13,14], which belong to the large family of G-protein coupled receptors that have seven transmembrane segments. Based on their pharmacological properties and structural homology, dopaminergic receptors are classified into two families consisting of D1-like receptors, which include the D1- and D5-receptors, and D2-like receptors, which include the D2-, D3- and D4-receptors [15].

The olivocochlear efferent neurons in guinea pig show tyrosine hydroxylase immunoreactivity, which is the enzyme that catalyzes the synthesis of DA [16]. DA is present in the cochlea at birth in the rat and its concentration increases with age to approximately 5-fold by 30 days after birth [17]. Sound conditioning triggers an up-regulation of tyrosine hydroxylase both in the lateral efferent of cochlea and in the lateral superior olivary complex and acoustic trauma reduced these levels [18].

DA modulates the sound evoked compound action potential (CAP) of auditory nerve with no effects on cochlear microphonic, summating or endocochlear potentials [19], indicating that DA action is taken place at postsynaptic level upon the terminals of afferent dendrites. The action of DA in the cochlea has been associated with a neuroprotective mechanism in afferent neurons, and the CAP modulation depends on the subtype(s) of DA receptors activated [20]. DA was shown to reduce the action potential amplitude in isolated SGNs of the mouse [21]. In the guinea pig, DA decreased the action potential discharge of afferent neurons that is induced by glutamatergic agonists in a dose-dependent manner [22]. The D1 receptor was localized at the spiral ganglia neurons and at the base of the IHC. The amplitude of the CAP was enhanced by D1 receptor agonists an effect that was abolished by a protein kinase A (PKA) inhibitor [23], and the level of glutamate receptor phosphorylation was increased by D1 receptor activation indicating that it is mediated by PKA signal transduction pathway [23]. Studies in mice have shown that D1 and D5 deletions reduce the response threshold to high frequency stimulation and that D2 receptor deletion increases the threshold for all frequencies. Mice with combined deletions of D2, D4 and D5 receptors show increased noise vulnerability [14].

In this work, we studied the effect of DA receptor pharmacology on the voltage gated I_Na_ of cochlear afferent neurons. We showed that down regulation of voltage-gated Na^+^ current by a second messenger cascade activated by DA receptors involves G proteins. The action of D1- and D2-like DA receptors was shown to be mediated by a G_αs_/AC/cAMP/PKA and G_αq_/PLC/PKC pathways respectively and may significantly contribute to neuroprotective action by regulating the gain of the afferent neurons.

## Material and Methods

The study was performed in strict accordance with the recommendations in the *Guiding Principles in the Care and Use of Vertebrate Animals in Research and Training* of the American Physiological Society and with the regulations of the *General Health Law Research Subject Health* of the Ministry of health of México. The animal protocol was reviewed and approved by the Institutional Animal Care and Use Committee (IACUC) of the Autonomous University of Puebla (VIEP-BUAP). All efforts were made to minimize animal suffering and to reduce the number of animals used. The animals were provided by the 'Claude Bernard' animal facility of the Autonomous University of Puebla.

### Isolation and culture of SGNs

For the animal experiments, Long Evans rats (postnatal day 8–9) were killed by decapitation. The upper part of the skull and the brain were removed, and the temporal bones were dissected from the cranium under a stereoscopic microscope (Nikon, Tokyo, Japan) and placed in L-15 medium (GIBCO, Grand Island, NY, USA). The bony shell of the cochlea, the stria vascularis and the organ of Corti were removed. The spiral ganglion was then carefully extracted from the cochlear modiolus and the bony spiral lamina. The spiral ganglion was incubated with 1.25 mg/ml collagenase IA and 1.25 mg/ml porcine trypsin dissolved in L-15 culture medium for 30 min at 37°C. The ganglia were then rinsed with fresh culture medium, triturated with a fire-polished Pasteur pipette, and centrifuged at 4,000 rpm for 5 min. The supernatant was discarded, and this procedure was repeated three times. Isolated neurons were plated on cover slides pretreated with 100 μg/ml poly-D-lysine (Sigma-Aldrich, St. Louis, MO, USA) in 35-mm petri dishes (Corning, Lowell, MA, USA) with 4 ml of modified L-15 medium (supplemented with 10% fetal bovine serum, 500 IU penicillin, 15.7 mM NaHCO_3_, 15.8 mM HEPES and pH adjusted to 7.4). The neurons were maintained in an atmosphere of 95% air and 5% CO_2_ at 37°C for 18–24 hours until recording, at which time the cover slides were mounted on the stage of an inverted phase-contrast microscope (TMS, Nikon Co. Tokyo, Japan) [24].

### Drugs

Drug perfusion was made with a gravity-driven flow system (flow rate of 0.5 ml/s) consisting of three square perfusion tubes coupled to a step motor (SF-77B; Warner Instruments, Hamden, CT, USA) for rapid solution change. DA, A-68930 (D1-like selective agonist), **SCH-23390** (D1 selective antagonist), quinpirole (D2-like agonist), eticlopride (D2-like antagonist), 8-Br-cAMP (cAMP analog), H-89 (PKA inhibitor), NPC-15437 (selective protein kinase C inhibitor), GDP-β-S (G protein unspecific blocker), U-73122 (Phospholipase C-PLC- inhibitor), forskolin (adenylyl cyclase activator), IBMX (cAMP phosphodiesterase inhibitor), Rp-cAMP (specific PKA inhibitor), NiCl_2_ (T type Ca^2+^ channel blocker), nifedipine (L type Ca^2+^ channel blocker), pertussis toxin (PTx, G_i/o_ protein irreversible inhibitor), and TTX (Na^+^ channel blocker) were all purchased from Sigma-Aldrich (St Louis, MO, USA). Dihydrexidine (D1 selective agonist) and okadaic acid (phosphatase 1 and 2A inhibitor) were purchase from Tocris Bioscience, (Ellisville, MO, USA) and Santa Cruz Biochemicals respectively. All drugs were prepared according to specifications. DA was always added with 100 μM of ascorbic acid. With some drugs, the experiments were performed in low light conditions.

The DA agonists and antagonists (DA, dihydrexidine, A-68930, quinpirole, SCH-23390, ketaserin, eticlopride) and the (8-Br-cAMP + IBMX, forskolin and okadaic acid) were applied in the bath in the extracellular solution for 2–4 min up to the stabilization of its effect. In some of the experiments such as those in which antagonists SCH-23390 and eticlopride were used, the drug was applied in the control recording (for 3 min) and then the capability of these drugs to block the action of an agonist was evaluated. Some drugs (GDP-β-S, H89, NPC-15437 and okadaic acid) were dissolved in the intracellular solution (2 min were allowed after establishing the whole cell for the drug to dialyze into the cell). In the experiments in which Rp-cAMP or U-73122 were used the cells were incubated with the drug for 30–60 min before the recording. For the experiments using PTx cells were incubated for 24 hrs with the toxin before recording.

### Electrophysiological recording

For electrophysiological recordings, the voltage and current clamp techniques in the whole-cell configuration were used. Experiments were performed at room temperature (23–25°C). For the voltage clamp, the cells were bathed with an external solution containing (in mM): 1.8 CaCl_2_, 1 MgCl_2_, 10 HEPES, 90 NaCl, 45 TEA-Cl, 10 4-AP and 10 glucose (at pH 7.4). For electrophysiological recordings, glass pipettes with a resistance of 2 to 5 MΩ were used, and the pipettes were pulled from borosilicate glass capillaries using a laser puller (P 2000, Sutter Inst. Novato, CA, USA). The pipette solution contained (in mM): 5 HEPES, 8 EGTA, 10 NaCl, 10 TEA-Cl, 30 CsCl, 100 CsF, 2 ATPMg and 1 GTPNa (at pH 7.2). Ionic currents from SGNs were recorded with an Axopatch 200B amplifier (Molecular Devices, Union City, CA). Command pulse generation and data sampling were performed with a 16-bit data acquisition system (Digidata 1440A, Molecular Devices, Union City, CA, USA) controlled by pCLAMP 10 software (Molecular Devices). Data were sampled at 10 kHz and low-pass filtered at 20 kHz. The passive properties of the cell, membrane capacitance (Cm), membrane resistance (Rm), access resistance (Ra) and time constants (Ƭ), were measured online with the pCLAMP program at −70 mV, with a 10 mV hyperpolarizing pulse. Capacitance and series resistance (80%) were compensated electronically.

Current-voltage relationships and availability curves were constructed using a standard double-pulse protocol; from a holding potential of −100 mV, a series of 50 ms prepulses between −110 and 50 mV were followed by a 50 ms test pulse to −20 mV (time interval between sweeps was 6 s). In all cases, the amplitude of the current, the half activation or inactivation voltage, and the respective slopes were evaluated by fits to a Boltzmann equation:

$$f\left(x\right)\;=\;\frac{A_{1}-A_{2}}{1+\;e^{\left(V-V_{1/2}\right)/dx}}+\;A_{2}$$

where *V*_*1/2*_ is the half-maximum voltage, *dx* is the slope factor, *A*_*1*_ is the minimum value, *A*_*2*_ is the maximum value, and *f(x)* is the probability.

The concentration-response curve was fit using a Hill equation:

$$E\;=\;\frac{E_{max}C^{n}}{{EC}_{50}^{n}+C^{n}}$$

where E is the predicted effect of the drug, *E*_*max*_ is the maximum effect, *C* is the drug concentration, *n* is the Hill coefficient and *EC*_*50*_ is the drug concentration producing half of the maximum effect.

For the current clamp experiments, a normal external solution of the following composition was used (in mM): 140 C_2_H_5_O_4_SNa, 1.8 CaCl_2_, 5.4 K-Gluconate, 1.2 MgCl_2_ and 10 HEPES. The internal solution contained (in mM): 10 C_2_H_5_O_4_SNa, 0.134 CaCl_2_, 125 K-Gluconate, 5 HEPES and 10 EGTA. Square current pulse series from −0.1 to 0.6 nA with 0.1 nA steps and 100 ms pulse lengths was used to determine the action potential threshold. The characteristics of the action potentials, elicited by 3 ms suprathreshold pulses, were analyzed off-line using Clampfit in the pClamp 10.2 bundle (Molecular Devices) and Origin 8.0 (Microcal Software, Northampton, MA, USA) software. The phase-plane plot of the response to current clamp pulses was constructed by plotting the first derivative of the membrane voltage with respect to the time (dV/dt) of the first action potential versus the membrane potential [25]. The threshold of the action potential was defined as the voltage at which dV/dt increases suddenly, and its amplitude was defined as the voltage between the maximum peak and the resting potential. The duration of the action potential was measured at 50% of the spike amplitude. The maximum depolarization rate (MDR) and maximum repolarization rate (MRR) were measured in the phase plane as the maximum and minimum dV/dt values, respectively. The afterhyperpolarization (AHP) was defined as the difference between the minimum voltage following the action potential and the membrane potential. Significant differences between the means were determined using Student’s t-test, and a value of *P* < 0.05 was considered statistically significant.

To study the drug effects in a more dynamical condition, sinusoidal current injection was used. The frequency of the stimuli was 10 or 20 Hz and cells were stimulated by sinusoidal current injection during 9 s, the amplitude was suprathreshold, based on the threshold value defined for each neuron by square current pulse injection described above. Values of current amplitude injection ranged from 150 pA to 600 pA.

Recordings were analyzed off-line using Clampfit 10 (Molecular Devices) and OriginPro 8 software (Microcal Software, Northampton, MA, USA). Statistical significance was determined using a paired Student’s *t*-test with *P* < 0.05. Numerical data are presented as the mean ± S.E.M. For comparisons between groups of different experimental series, unpaired *t*-test was used.

## Results

### DA effect on the I_Na_ amplitude of SGNs

**Fig 1:**
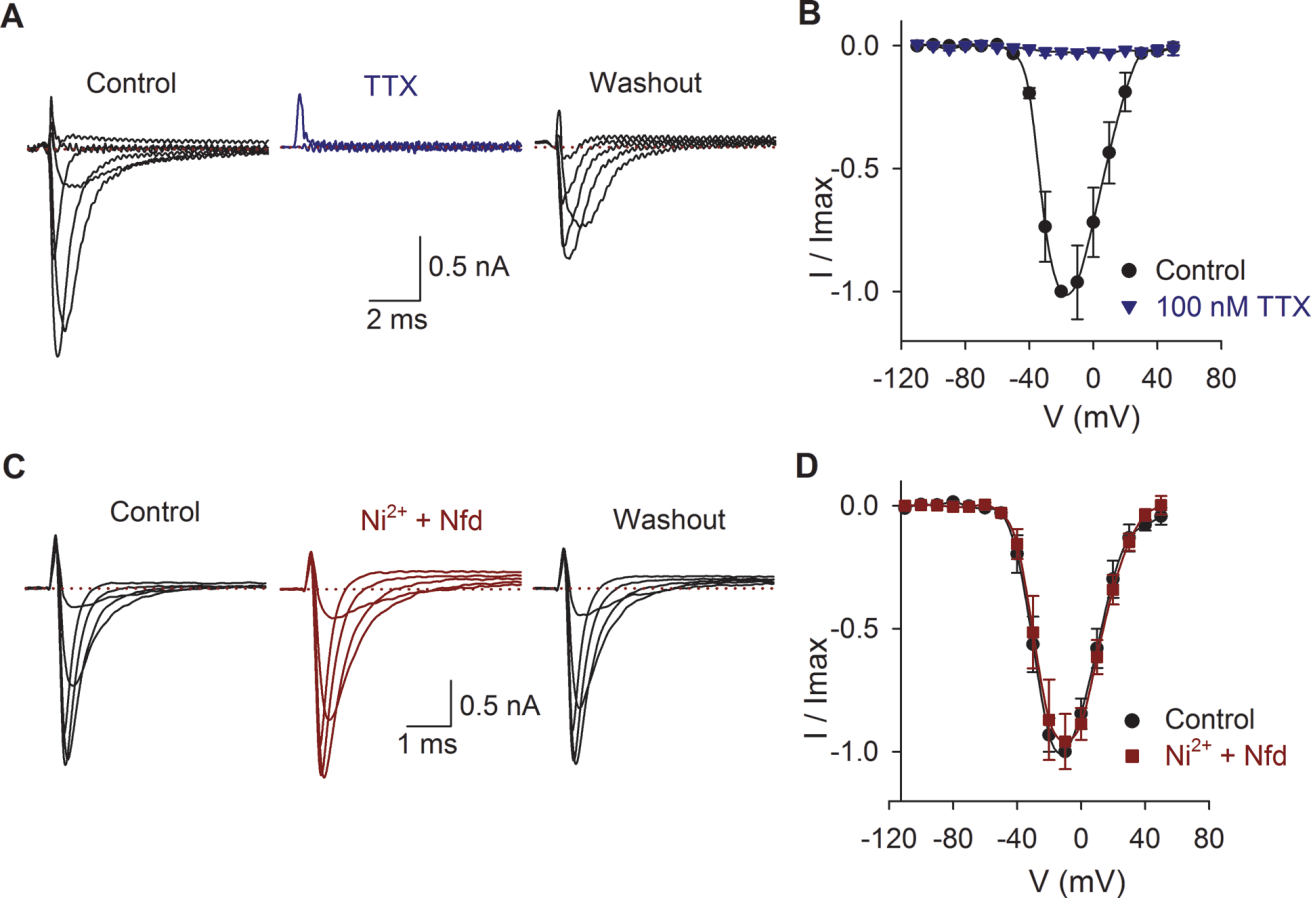
Effects of TTX, nickel and nifedipine on the I_Na_. A) I_Na_ produced by voltage pulses from −110 mV to 50 mV in control conditions, during 100 nM TTX perfusion and 2 min after washout. In this and the following figures, only representative traces are shown. B) Current-voltage relationship in control conditions (black) and with TTX perfusion (blue). C) Current recording in control conditions and after coapplication of 10 μM nifedipine (Nfd) and 100 μM nickel. D) Current-voltage relationship in control conditions (black) and with Ni^2+^ plus nifedipine perfusion (red). In all traces, the dotted line indicates zero current.

The average capacitance of SGNs (*n* = 380) was 9 ± 0.5 pF. Some cells were identified as basal or apical cells, and no difference in membrane capacitance was found between cells from basal (8 ± 0.4 pF, *n* = 52) or apical portion of the cochlea (8 ± 0.3 pF, *n* = 85). The I_Na_ was blocked by TTX (Fig. 1A-B) and unaltered by the calcium channel antagonists nifedipine (10 μM) and nickel (100 μM) (Fig. 1C-D). The average density of the sodium current was 216 ± 13 pA/pF (*n* = 97). No correlation between the current amplitude and the cell membrane capacitance was found (R^2^ = 0.01).

**Fig 2:**
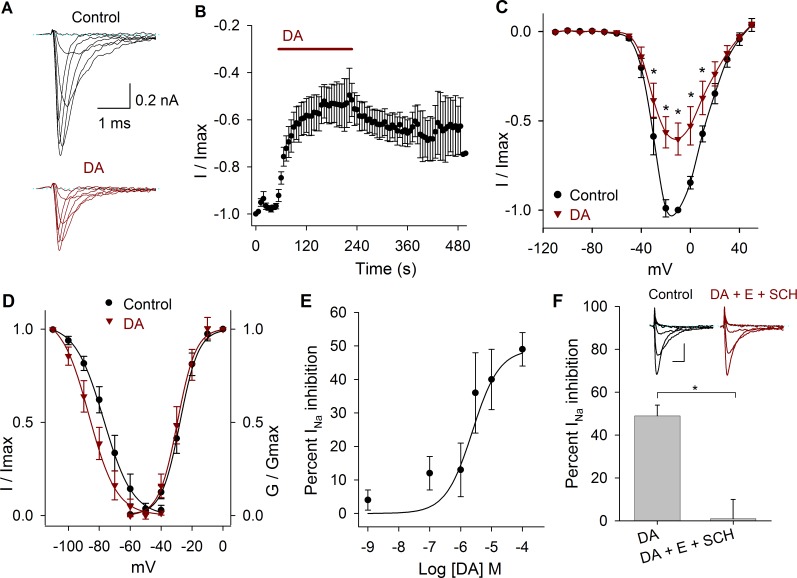
Effects of DA on the I_Na_. A) Representative experiment showing the effect of 10 μM DA after control perfusion. B) Temporal course of the inhibitory action of DA 100 μM on the I_Na_ amplitude. Bar indicates the DA perfusion. C) Current-voltage relationship in control and with 10 μM DA. DA decreased the I_Na_ by 43 ± 8% at −20 mV (*P* = 0.001; *n* = 9). D) Activation and inactivation curves in control and after DA application. DA caused a hyperpolarizing shift of the V_½_ of the inactivation curve of 8 mV at 10 μM (*P* = 0.8). In this and following activation and inactivation curves the data were fitted with a Boltzmann function (solid lines). E) Concentration-response relationship of the effect of DA (1 nM to 100 μM), with at least *n* = 6 for each point. The data were fitted with a concentration response curve (solid line) with an IC_50_ of 2.5 x 10^–6^ M and a Hill coefficient of 1. F) Bar graph shows that a mixture of D1 and D2 antagonists (100 μM SCH-23390 + 1 μM eticlopride) completely blocks DA action (*P* = 0.006). Inset show a representative recording of the SCH-23390 (SCH) and eticlopride (E) actions. Calibration bars 0.2 nA and 1 ms. Asterisks denote a significant effect *P* < 0.05.

Perfusion of DA reduced the I_Na_ peak amplitude, an effect that took place during the first minute of its application (Fig. 2A-B). Comparing the values of the current amplitude at −10 mV, DA significantly decreased the Na^+^ current at 3 μM, 10 μM and 100 μM by 36 ± 12%, 40 ± 9% and 49 ± 5%, respectively, and shifted the V_1/2_ towards more negative potentials. When 10 μM of DA (*n* = 9) was perfused, the maximum I_Na_ amplitude decreased 43 ± 8%, *P =* 0.001 at −20 mV (Fig. 2C). DA caused a significant 7 mV, 12 mV, 8 mV, 9 mV and 14 mV hyperpolarizing shift in the V_½_ of the inactivation curve at 1 nM (*P* = 0.03), 100 nM (*P* = 0.001), 1 μM (*P* = 0.02), 3 μM (*P* = 0.01) and 100 μM (*P* < 0.001), respectively. With 10 μM DA non-significant changes of the activation or inactivation curves was found (*n* = 9; *P* > 0.05) (Fig. 2D). The concentration-response curve of the effect of DA on the I_Na_ showed that DA inhibits the I_Na_ in a concentration dependent manner (Fig. 2E). Data were fitted (solid line) by a dose-response function with an IC_50_ of 2.5 μM.

The effect of 100 μM of DA on the peak I_Na_ amplitude was blocked by the co-application of 100 μM SCH-23390 and 1 μM eticlopride (D1- and D2-like antagonists). In control DA produced an inhibition of the I_Na_ of 49 ± 5% (*n* = 6; *P* < 0.001), and after the coapplication of antagonists its effect was reduced to 1 ± 9% (*n* = 5, *P* = 0.92, Fig. 2F).

### G protein and Na^+^ channel phosphorylation

**Fig 3:**
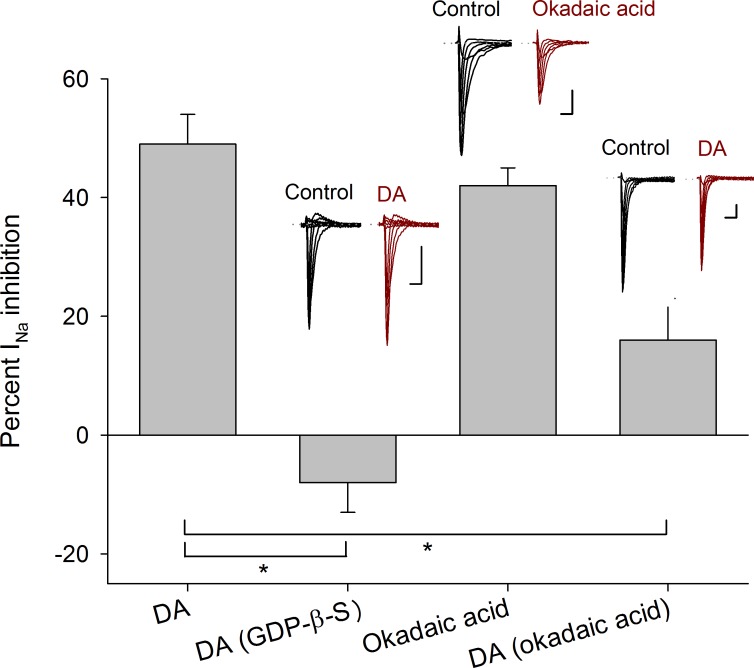
Intracellular mechanism inherent to D1- and D2-like receptors. Bars indicate the percent I_Na_ inhibition produced by 100 μM DA and compared with its effect when 500 μM GDP-β-S (*P* < 0.001) were added intracellularly. The perfusion of 100 nM okadaic acid decreased the I_Na_ amplitude in a similar percentage as 100 μM DA (*P* = 0.3 unpaired *t-test*). While 100 nM okadaic acid added intracellularly occluded the DA action (*P* = 0.01). Insets above bars show representative recordings in control conditions and after drug application. Asterisks denote a significant difference (*P* < 0.05 unpaired *t-test*). Calibration bars are 1 ms and 0.5 nA for all recordings.

To determine the participation of G-proteins in the DA action, GDP-β-S (500 μM), which is a non-hydrolyzable GDP analog was used to block G proteins (GDP-β-S was dissolved in the intracellular solution, *n* = 5). In this condition, DA (100 μM) perfusion did not produce any significant effect on the I_Na_ amplitude (current increased 8 ± 5%, *P* = 0.265), nor significant changes in the activation or inactivation curves or voltage sensitivity of the I_Na_, indicating that DA receptor activation implies the activation of a G-protein coupled receptor (Fig. 3).

When the inhibitor of type 1 and 2A protein phosphatases, okadaic acid (100 nM), was perfused I_Na_ peak amplitude decreased 43 ± 2% (*n* = 5, *P* < 0.001) and shifted the half inactivation voltage towards more negative potentials (from −70 ± 1 mV to −80 ± 1 mV, *P* = 0.003) with not change of the slope. Non-significant changes of the activation curve were found (Fig. 3).

Since okadaic acid promotes the phosphorylated state of the sodium channels (assuming that, in the cells, the basal concentration of cAMP activates the PKA that phosphorylate them and PKA autophosphorylation on Thr^197^ site maintain the PKA in active state) the inclusion of 100 nM okadaic acid dissolved in the intracellular solution was tested (*n* = 5). Okadaic acid reduced the inhibition produced by 100 μM DA from 49 ± 5% under control conditions to 16 ± 7% (*P* = 0.126). With okadaic acid non-significant changes in the activation or inactivation curves or voltage sensitivity of the I_Na_ was produced by DA. These indicate that phosphorylation of the Na^+^ channel is the result of DA receptor activation (Fig. 3).

### Functional expression of D1-like receptors

**Fig 4:**
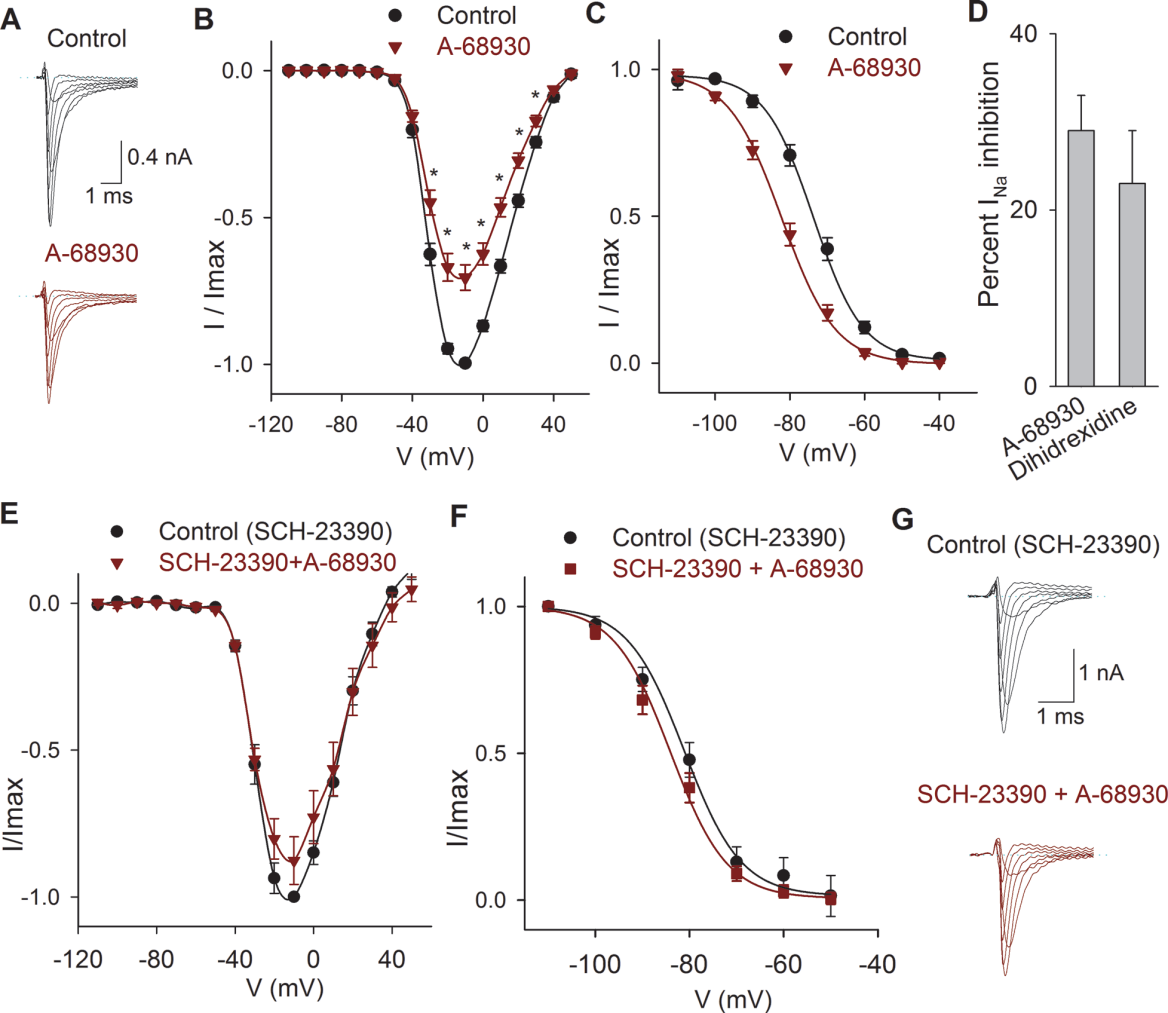
Effects of D1 related drugs on the I_Na_. A) Recordings of the I_Na_ in control condition and after perfusion of A-68930 (300 nM). B) Current-voltage relationship of the I_Na_ under control conditions and after 300 nM A-68930. The maximum decrease of the current was 29 ± 4% at −10 mV. C) Steady state inactivation of the I_Na_ in control conditions and after 300 nM A-68930, which caused a leftward shift of the inactivation. D). Bar graph comparing the inhibitory effect of 100 nM dihidrexidine with A-68930 effect. Non-significant difference was found (*P* = 0.445; unpaired *t-test*). E) Current-voltage relationship of the I_Na_ in control conditions (with 100 μM SCH-23390), and after the co-application of 300 nM A-68930. F) Steady state inactivation of the I_Na_ in control (with 100 μM SCH-23390) and after A-68930 + SCH-23390, which caused non-significant changes. G) Typical recordings of the I_Na_ showing that SCH-23390 significantly reduced the inhibitory action of 300 nM A-68930.

Two D1-like agonists were studied A-68930 and dihydrexidine. The perfusion of the D1-like agonist 300 nM A-68930 (*n* = 14) decreased the I_Na_ amplitude 29 ± 4% (*P* < 0.001) (Fig. 4A-B) and shifted the V_½_ of the inactivation curve 7 mV leftward (*P* < 0.001), with no change in the slope of inactivation (Fig. 4C). Experiments using the D1 agonist dihydrexidine (100 nM) shown that it also inhibits I_Na_ 23 ± 6% (*P* = 0.009, *n* = 5; Fig. 4D) and displaced the inactivation V_½_ 10 mV to more negative potentials (from −71 mV in control to-81 mV; *P* = 0.01).

Experiments in which the D1 antagonist SCH-23390 (100 μM) was perfused shown that this drug blocked the action of 300 nM A-68930 (*n* = 5) reducing its effect to a non-significant 12 ± 5% inhibition of the I_Na_ (*P* = 0.073) indicating that agonist action takes place by specific D1 receptor activation (Fig. 4E-G). Paradoxically, we found that the application of 300 nM of SCH-23390 significantly decreased the I_Na_ (between −30 mV and 40 mV) with a maximum effect of 31 ± 3% (*n* = 5, *P* = 0.001). The coapplication of A-68930 with SCH-23390 produced an additional inhibition of 29 ± 3% (*P* < 0.001). With 300 nM of SCH-23390, the V½ of the inactivation curve shifted 10 mV leftward (*P* < 0.001), and co-application with 300 nM of A-68930 shifted the inactivation curve an additional 4 mV leftward (*P* = 0.016) (Data not shown).

Ketanserin, a serotonin receptor antagonist, also behaves as a D1 and D5 receptors antagonist [26]. In our experiments when ketanserin (1 μM) was co-applied with A-68930 300 nM (*n* = 5), the agonist no longer produced a significant effect on the I_Na_ peak amplitude, the V_1/2_ of the inactivation curve was shifted from −66 ± 1 mV to −76 ± 1 mV (*P* = 0.01) without modification of the activation curve (Data not shown).

### Signaling pathway activated by D1-like receptors

To determine the D1 signaling mechanisms two pathways were considered: D1 coupled to G_αs_ protein and D1 coupled to G_αq_ protein.

**Fig 5:**
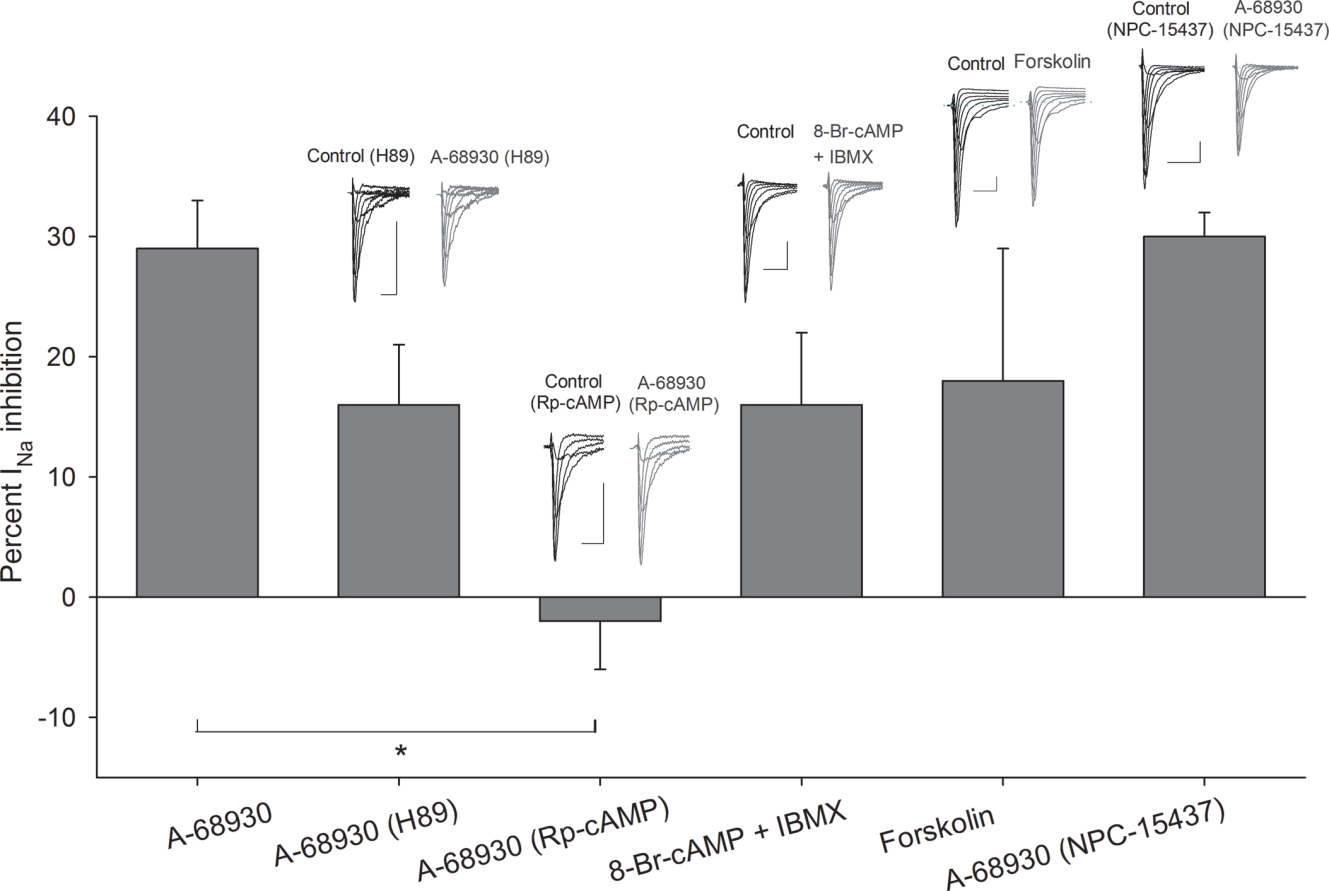
Transducer mechanisms activated by D1-like receptors. Bars show the effect of A-68930 in control condition in comparison with its action while other drugs were used. The use of H89 in the intracellular solution decreased the inhibitory effect of A-68930 from 29 ± 4% to 16 ± 5% at −10 mV (*P* = 0.08). When cells were preincubated with 50 μM Rp-cAMP, A-68930 effect was completely blocked (*P* < 0.001). Coapplication of 8-Br-cAMP and IBMX mixture caused a decrease in the I_Na_ current which mimicked the effect of A-68930 (*P* = 0.114). Forskolin also mimicked the effect of D1-like agonist decreasing the I_Na_ 18 ± 11% (*P* = 0.27). The NPC-15437 in the intracellular solution did not significantly modify the A-68930 effect (*P* = 0.92). Insets above bars show typical recordings of the I_Na_ under control conditions and after drug application. Calibration bars are 2 ms and 0.5 nA for all recordings.

Inclusion of a selective inhibitor of PKA, H89 (1 μM), in the intracellular solution (*n* = 6) produced a decrease in the effect of 300 nM of A-68930 on the I_Na_ from 29 ± 4% in control condition to 16 ± 5% (at −10 mV) (*P* = 0.027). No changes in the V_½_ or the slope of activation or inactivation curves were found (Fig. 5).

Preincubation of the cells with Rp-cAMP (50 μM, during 30–60 min), which is one of the most specific PKA inhibitors available, completely blocked the action of 300 nM A-68930 on the I_Na_ (current increased 2 ± 4%, *P* = 0.644). No changes in the V_½_ or the slope of activation or inactivation curves were observed. These results show that PKA participate in the signaling pathway activated by D1-like receptor (Fig. 5).

An experimental series using 8-Br-cAMP (500 μM), along with cAMP phosphodiesterase inhibitor IBMX (100 μM), produced a 16 ± 6% (*P* = 0.04, *n* = 5) inhibition of the I_Na_, with non-significant changes in the activation or inactivation curves or voltage sensitivity of the I_Na_ (Fig. 5). Furthermore, to determine the adenylyl cyclase (AC) participation in the pathway activated by D1-like receptors, forskolin (AC activator) was used. Forskolin (10 μM) decreased the I_Na_ 18 ± 11% (*n* = 5: *P* = 0.04). The V_½_ of the inactivation curve shifted 8 mV leftward (*P* = 0.02; Fig. 5). Together, these results indicate that intracellular increments of cAMP produce by itself a significant decrease of the I_Na_ mimicking the D1-like receptor activation.

The use of 500 nM NPC-15437 (selective PKC inhibitor) in the pipette produced non-significant changes in the inhibitory action of 300 nM A-68930 (*n* = 8), which decreased the I_Na_ 30 ± 2 at −20 mV (*P* < 0.001) (Fig. 5). The V_½_ and slope of the activation curve did not change, and the V_½_ of the inactivation curve shifted 12 mV leftward (*P* < 0.001) with no change of the slope (Fig. 5). This result indicates that PKC is not significantly participating in the D1-like signaling pathway.

### Functional expression of D2-like receptors

**Fig 6:**
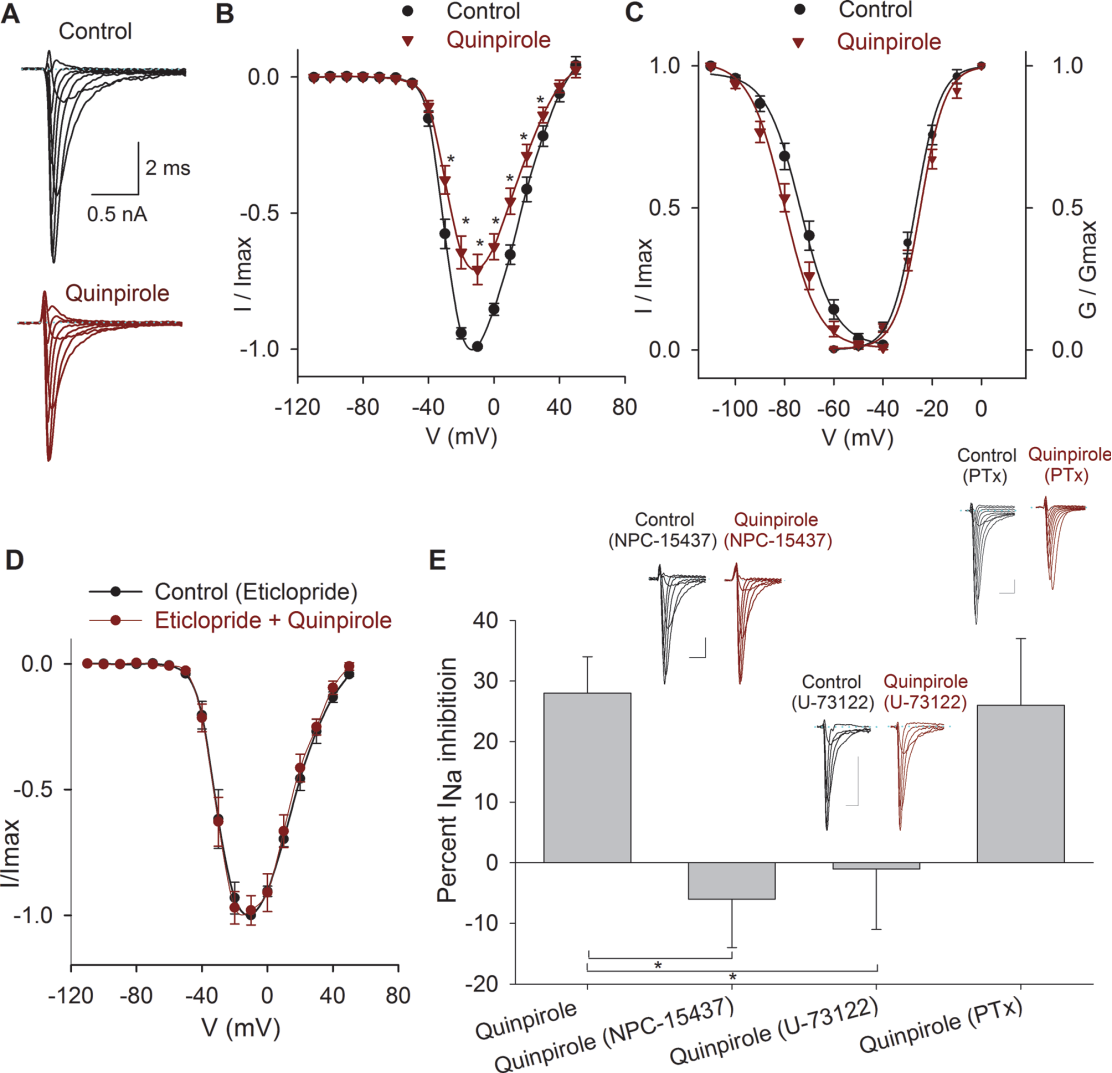
Effects of quinpirole on the I_Na_. A) Typical recordings of the I_Na_ under control conditions and after 1 μM quinpirole perfusion. B) Current- voltage relationship of the I_Na_ in control conditions and after 1 μM quinpirole. The maximum inhibition was 28 ± 6% at −10 mV. C) Conductance activation and steady state inactivation curves of the I_Na_ in control conditions and after 1 μM quinpirole perfusion. Quinpirole caused a 5 mV hyperpolarizing shift in the V_½_ of the inactivation curve. D) Current-voltage relationship of the I_Na_ in control condition (with 1 μM eticlopride) and after coapplication of 1 μM quinpirole. E) Bar graph of the percent inhibition of the I_Na_ by quinpirole in control, with NPC-15437 in the intracellular solution (*P* = 0.004) and when the cells were preincubated with U-73122 (*P* = 0.02) or PTx (*P* = 0.82). Insets above bars show typical recordings of the I_Na_ in control and after drug application. Calibration bars 0.5 nA, 1 ms for all recordings.

The application of the D2-like receptor agonist quinpirole (1 μM) significantly decreased the I_Na_ peak amplitude at all the voltages (*n* = 10) (Fig. 6A-B). At −10 mV, the I_Na_ peak amplitude was 28 ± 6% (*P* = 0.001). The V_½_ of the inactivation curve was shifted 5 mV leftward (*P* < 0.001), with no changes in the slope (Fig. 6C). The effects of quinpirole were blocked by 1 μM eticlopride (D2-like receptor antagonist), which reduced quinpirole action to 2 ± 6% (*n* = 4; *P* = 0.76, Fig. 6D).

### Signaling pathway activated by D2-like receptors

To study the signaling mechanisms of D2-like receptors two possibilities were considered: D2 receptors coupled to G_αq_/PLC/PKC protein or D2 receptors coupled to G_i/o_ protein.

Initially, the selective PKC inhibitor NPC-15437 (500 nM) dissolved into the pipette solution was tested when activating the D2 receptors with quinpirole (1 μM, *n* = 5). NPC-15437 produced a significant block of D2 agonist effect whose action becomes under NPC-15437 a non-significant increase of 6 ± 8% of the I_Na_ (*P* = 0.5); thus indicating that D2 receptors are coupled to a signaling pathway that leads to PKC activation (Fig. 6E). In cells that were preincubated for 30–60 min with 10 μM U-73122 (PLC blocker), the subsequent perfusion of 1 μM quinpirole produced a non-significant 1 ± 10% increase of the I_Na_ (*n* = 6, *P* = 0.91) (Fig. 6E).

When cells were pretreated (for 24 hrs) with 400 μM of PTx, quinpirole inhibition of the I_Na_ was 26 ± 11% (*n* = 5, *P* = 0.027) which is similar to the inhibition of 28 ± 6% produced under control condition (*P* = 0.001) (Fig. 6E). Together these results indicate that D2-like receptor activation involves G_αq_/PLC/PKC pathway.

### Current Clamp Experiments

**Fig 7:**
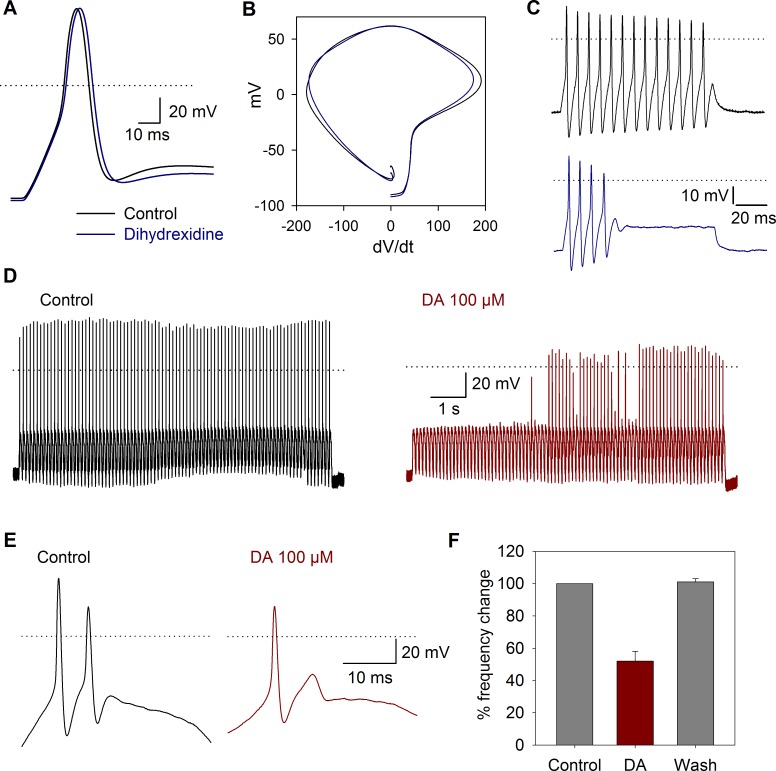
Current clamp response to sinusoidal stimulation and DA receptor activation. A) The use of 100 nM dihydrexidine (blue traces) shifted the maximum repolarization rate and increased the action potential duration. B) Phase plane plot of action potentials under control conditions and after dihydrexidine application. C) Action potentials produced by a current pulse injection of 150 pA were reduced from 13 under control conditions to 4 after dihydrexidine (100 nM). D) Typical response to sinusoidal current injection (10 Hz, 150 pA). Before the stimuli, the cells were held at −80 mV. E) In a cell discharging in a 2:1 phase lock to sinusoidal stimuli, the use of DA reduced action potential discharges per cycle to a phase lock of 1:1 F) Bar graph showing the percent change in the number of action potentials in the control, after 100 μM DA application and after washout of the drug.

Current clamp recordings of SGNs were also performed to examine voltage responses to current pulse injection. The cell voltage was fixed at −80 mV, and at more depolarized membrane voltages, no action potential discharge was produced even by highly depolarizing (> 50 mV) current pulse injection. Action potential waveform parameters under control conditions were (*n* = 24): amplitude = 117 ± 5 mV, duration = 1 ± 0.2 ms, latency = 4 ± 1 ms, threshold = -38 ± 2 mV, MDR = 144 ± 11 mV/ms, MRR-150 ± 9 mV/ms. The use of DA agonists in current clamp experiments revealed that action potential parameters showed various modifications, summarized in Table 1. DA 100 μM decreased the action potential amplitude 10 ± 3%, (*n* = 4, *P* = 0.04) with non-significant changes in other action potential parameters. Under 100 ms current injection, 90% (*n* = 46) of the cells produced a single action potential when stimulated with suprathreshold depolarizing current pulses. The other 8% slowly adapted producing between 3–7 action potentials, and only 2% of the cells produced sustained, non-adapting, repetitive spiking. In neurons with repetitive spike activity under square current pulse injection, the D1 agonist dihydrexidine (100 nM, *n* = 10) produced a significant decrease of the MRR from −154 to −140 mV/ms (*P* = 0.005). The duration at 50% of the action potential was increased by 7 ± 0.1% (*P* = 0.005), and in two cells that showed repetitive spike activity, a reversible (44%) reduction in the number of action potentials produced by the current pulse injection was induced (Fig. 7A-C).

**Table 1:** Action potential parameters in control conditions and after drug use.

Drug	Parameter	Control	Effect	P-value	Tendency
A-68930 (300 nM) n = 4	Threshold (mV)	-25 ± 1.5	-24 ± 3.32	0.76	↓
	Amplitude (mV)	132 ± 7	137 ± 11	0.3	↑
	MDR (mV/ms)	140 ± 23	137 ± 24	0.82	↓
	MRR (mV/ms)	-124 ± 15	-111 ± 19	0.12	↓
	Latency (s)	0.9 ± 0.04	1 ± 0.03	0.22	↑
	Duration (s)	2 ± 0.3	2.2 ± 0.4	0.18	↑
Quinpirole (1 μM) n = 4	Threshold (mV)	-40 ± 6	-46 ± 4	0.4	↑
	Amplitude (mV)	88 ± 7	88 ± 6	0.83	↓
	MDR (mV/ms)	144 ± 43	131 ± 33	0.42	↓
	MRR (mV/ms)	-154 ± 33	-144 ± 23	0.4	↓
	Latency (s)	0.5 ± 0.2	0.4 ± 0.1	0.15	↓
	Duration (s)	1.6 ± 0.3	1.7 ± 0.3	0.06	↑
A-68930 (300 nM) + quinpirole (1 μ) n = 6	Threshold (mV)	-42 ± 4	-42 ± 4	0.94	↓
	Amplitude (mV)	129 ± 7	125 ± 8	0.22	↓
	MDR (mV/ms)	146 ± 24	130 ± 23	0.28	↓
	MRR (mV/ms)	-157 ± 22	-149 ± 19	0.45	↓
	Latency (s)	1.2 ± 0.1	1.2 ± 0.1	0.57	↑
	Duration (s)	1.8 ± 0.2	1.8 ± 0.2	0.39	↑
Dihydrexidine (100 nM) n = 10	Threshold (mV)	-41 ± 2	-40 ± 1.6	0.52	↓
	Amplitude (mV)	116 ± 6	114 ± 9	0.16	↓
	MDR (mV/ms)	144 ± 17	272 ± 147	0.4	↑
	MRR (mV/ms)	-154 ± 13	-140 ± 14	* 0.005	↓
	Latency (s)	1.7 ± 0.1	1.7 ± 0.1	0.25	↑
	Duration (s)	0.5 ± 0.1	0.5 ± 0.1	* 0.005	↑

To study the dynamics of the action potential discharge suprathreshold sinusoidal current pulses were used. The sinusoidal stimulation showed that application of 100 μM DA (*n* = 7) produced a modulation of SGN excitability, significantly reducing the spike discharge induced by the 10 or 20 Hz sinusoidal stimuli (*P* = 0.001; Fig. 7D-F).

## Discussion

The SGN consist of 95% type I, and 5% type II cells [27], thence it is 19 times more likely to encounter a type I cell. Additionally, the capacitance of the cells in our experiments coincided with the value reported for type I cells (9 ± 0.5 pF, n = 380). The type II neurons have lower capacitance ≅ 6 pF [28], and they are not innervated by dopaminergic fibers [29]; thus, altogether these data support the notion that we were recording essentially from type I neurons.

DA-receptor gene expression studies in the cochlea have shown significant interspecies differences. Transcripts of the genes for D2 (long) and D3 receptors were found in SGNs from the mouse cochlea (14–18 days old), while those of the D2 (Short) and D4 receptors were not amplified [12]. In contrast, all DA receptor subtypes were found in rat SGNs [13]. A recent report using immunohistochemistry and RT-PCR in postnatal day 10–13 mice described the expression of D1, D2 and D5 receptors in SGNs and OHC, and D4 receptor was found exclusively in SGN. With no evidence of D3 receptor expression in mice cochlear tissues [14].

Our results show that DA, A-68930 and quinpirole, cause a decrease of the I_Na_ in SGNs. The D2-like antagonist eticlopride blocks the effect of quinpirole, indicating the specific action of quinpirole and the functional expression of D2-like receptors. The D1-like antagonist SCH-23390 blocks the action of A-68930, indicating the functional expression of D1-like receptors in the spiral ganglion neurons. The use of ketanserin shows that it blocks the A-68930 action on the I_Na_ amplitude, thus behaving as a D1-like receptor antagonist. It is worth noting that SCH-23390 and eticlopride have an inhibitory effect on the I_Na_ by itself. A similar paradoxical effect of these antagonists was observed in the auditory activity of guinea pig [19]. The intrinsic inhibitory action of the antagonist may be produced because DA receptors may have constitutive activity similar to that found in histamine receptors [30] and in D1/D2 receptor chimeras [31]. There is also a group of D1-like receptors that are insensitive to the antagonism of SCH-23390 [32]. The inhibitory effect of SCH-23390 could also be due to an unspecific interaction with other non-dopaminergic receptors or due to a direct effect on ionic channels.

To determine the intracellular pathways participating in the dopaminergic action in the SGN, we employed a pharmacological approach. Use of GDP-β-S decreased the DA effect, indicating that DA receptor actions are mediated by a G protein. In most cell types in which a G protein modulation of the I_Na_ has been described, the most common mechanism was a reduction of the current amplitude due to a decrease in the Na^+^ channel availability [33], although also a negative adjustment of the voltage dependence of rapid inactivation has been described [33]. In our experiments, both mechanisms seem to be occurring since we found current amplitude reduction and modifications of the inactivation V_½_ with most of the drugs tested. Considering that okadaic acid reproduces and occludes the DA effect, we concluded that Na^+^ channel phosphorylation and subsequent modification of the current amplitude is essential for the DA action.

The D1-like and D2-like receptors act through different pathways. In our system, blocking PKA with H89 or Rp-cAMP significantly reduced the action of the D1-like agonist, indicating that PKA participates in its signal transduction. Previous studies using H89 and forskolin have also shown that the dopaminergic modulation of the CAP in the auditory system implies the PKA participation [23].

**Fig 8:**
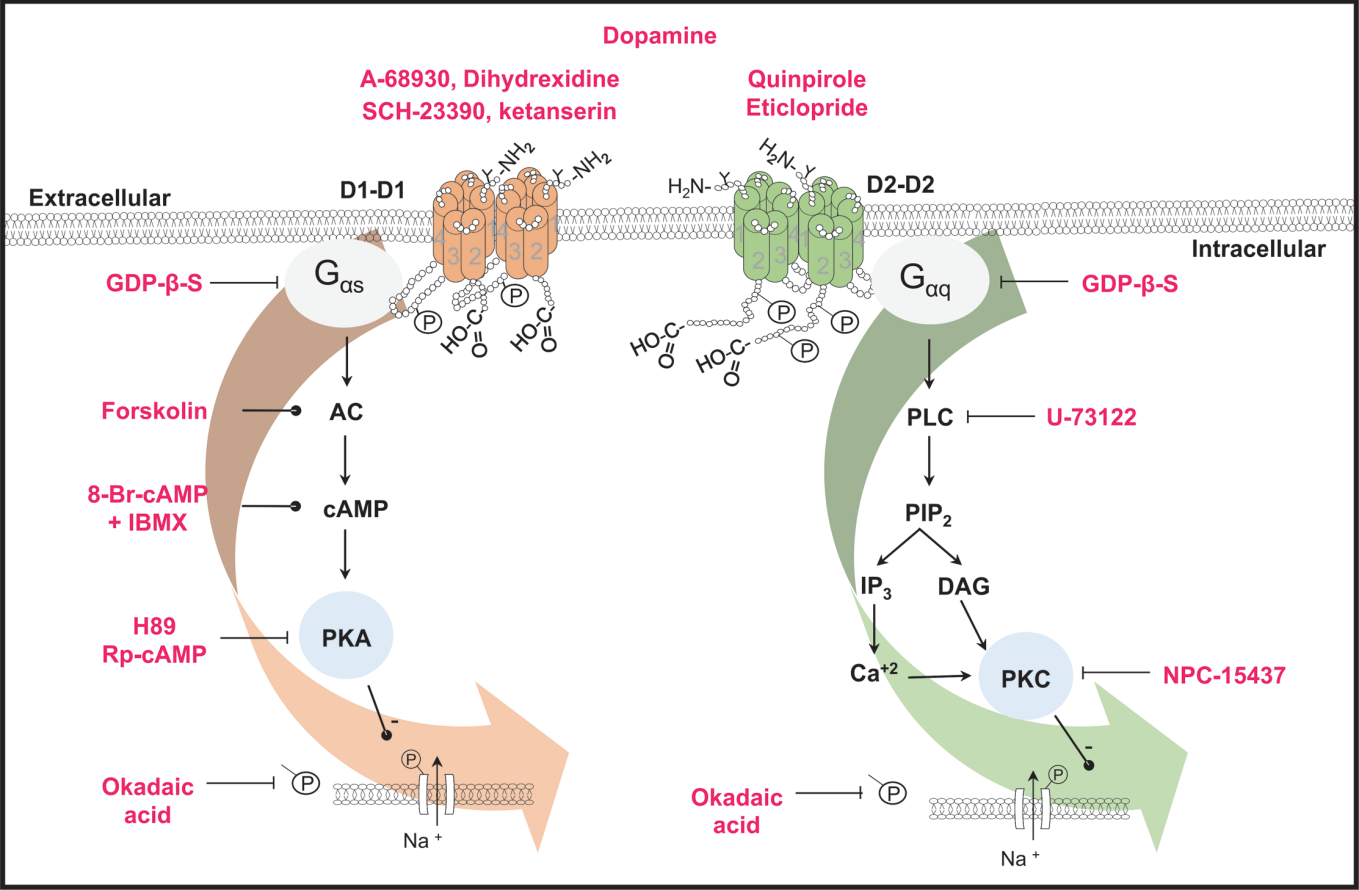
Scheme of the signaling pathways activated by D1- and D2-like receptors in the spiral ganglion neurons. Receptors are shown as homodimers. Phosphorylation and glycosylation sites are indicated (P and Y). The drugs used in this work are shown in red. Lines with transverse-endings indicate blockade and those with circle-endings stimulation. D1-like receptors are coupled to a G_αs_ protein leading to AC stimulation, thus increasing cAMP levels and subsequent PKA activation. PKA phosphorylates the Na^+^ channels thus decreasing the I_Na_. D2-like receptors are coupled to a G_αq_ protein whose activation stimulates the PLC, which cleaves PIP_2_ into IP_3_ and DAG, the IP_3_ increases the Ca^2+^ concentration, and both Ca^2+^ and DAG activates PKC leading also to a Na^+^ channel phosphorylation thus decreasing the I_Na_. In both cases, phosphorylation was prevented by okadaic acid.

Our results show that increasing the cAMP levels (using a cAMP analog plus a phosphodiesterase inhibitor or an AC activator) mimics the DA effect. Thus indicating that increase in the cAMP secondary to adenylyl cyclase activation leads to PKA activation. Together these results showed that D1-like dopamine receptors activate a G_αs_/AC/cAMP/PKA pathway (Fig. 8). However, we cannot rule out the involvement of additional signaling pathways. The D1-like receptors have been shown to be coupled to three different signaling mechanisms: SCH-23390 sensitive G_αs/olf_/AC/PKA, adenosine A2A receptor-dependent G_αs/olf_/AC/PKA and G_αq_/PLC [32]. In our experiments no significant effect was produced by PKC inhibition on D1-like receptor actions, indicating that Gαq protein seems not to participate in the SGNs D1 receptors response. In neostriatal neurons, even a marginal activation of PKC potentiates the effects of PKA [32], and in striato-nigral neurons (P25 rats), D1-like receptors decrease the I_Na_ via PKA, but PKC is also involved in the I_Na_ modulation [34].

In addition, protein-protein interactions of the carboxy terminal intracellular sites of the D1 receptor with N-methyl-D-aspartic acid (NMDA) receptor subunits [35, 36], and GABA-A interactions with the D5 receptor [37] have been described. The D1 and D2 heteromeric receptors may interact with A1 and A2A adenosine receptors [38]. Heterodimers of metabotropic glutamate receptors mGlu(5) and D2 receptors and oligomeric receptors containing more than two protomers, including mGlu (5), D2 and A2A adenosine receptors, are found in rat striatum homogenates [39]. Also heteromers of D1 with type 3 histamine receptor (H3) have been shown in transfected mammalian cells and confirmed by bioluminescence resonance energy transfer (BRET) and binding assays [40]. Thus a complex pharmacology of the D1 agonists and antagonists is not an unexpected phenomena given the complexity of receptor interactions with other membrane proteins.

The D2-like receptors may be coupled to G_αi/o_ or G_αq_ proteins. In striatal medium spiny neurons and cholinergic interneurons, the activation of D2 receptors leads to a potent decrease of the I_Na_ through a PKC-dependent mechanism [41]. In our system blockade of PKC eliminates the effect of the D2-like receptor activation. Additionally, block of Phospholipase C (PLC); which inhibits the hydrolysis of phosphatidylinositol 4,5-bisphosphate (PIP_2_) to inositol trisphosphate (IP_3_) and diacyl-glycerol (DAG), decreasing the cytosolic free calcium necessary for PKC activation, also produced a complete block of the effects due to D2-like receptor activation (Fig. 8). Since no significant effect of PTx was found, we concluded that in our system, the G_αi/o_ did not to have a role in reference to the I_Na_ current modulation and most probably D2-like signaling pathway is through G_αq_ /PLC/PKC pathway activation.

The activation of D1- or D2-like receptors exerts similar action on the I_Na_ in SGNs. It is possible that, in these cells, both DA receptors act in a complementary manner. The DA receptors exist as homomers, but they may also form heteromers, which has been demonstrated by co-immunoprecipitation. The heteromers often exhibit properties and signaling pathways different from those of their constituent receptors [42,43,44]. In striatal medium spiny neurons, the D1 and D2 receptors are segregated into discrete populations, but there is increasing functional and anatomical evidence that they may be co-expressed in a subpopulation of neurons. A new complex receptor in the striatum, the D1-D2 heteromeric receptor, with unique functional features would constitute a third neuronal population with physiological relevance [45].

In the inner ear, the complexity of the sound stimuli make it important to have a finely tuned system capable of segregating the features of each sound. Such process requires very precise differential modulation of the afferent neuron gain. In the cochlear efferent system, precision seems to be obtained thanks to the participation of various neurotransmitters including ACh, GABA, CGRP, enkephalins and DA, among others. We propose that the function of the release of multiple neurotransmitters from the efferent terminals and the expression of their receptors in SGNs is that all neurotransmitters act in a synergistic form, carrying specific functions to establish their final integrated effects

We found that under current pulse stimulation most of the SGN adapted quickly and discharged a single action potential. These results coincide with those described by other authors [46,47]. Applying DA to cells stimulated with sinusoidal current injection allowed us to show the inhibitory action of DA in the action potential discharge of SGNs. The use of dihydrexidine in cells with repetitive discharge showed that there is an increase in the duration of the action potential in the cells that discharged throughout the pulse. This change in the AP duration is reflected by a change in the number of action potentials throughout the current pulse. These results support the notion that DA released from the olivocochlear efferent neurons depresses the discharge of the afferent neurons, constituting a protective mechanism in conditions of ischemia or acoustic overstimulation, which may lead to an excitotoxic damage from excess glutamate release and sustained action potential discharge [48,49]. The DA neurotransmission enhancer rasagiline (a monoamine oxidase type B inhibitor) has been approved as an otoprotectant, showing that DA release from lateral olivocochlear fibers exert a protective action against excitotoxicity, a pathological factor in the aminoglycoside-induced sensorineural hearing loss [50]. Also pramipexole (a D2/D3 receptor agonist) is an effective agent against subjective tinnitus, action that may involve both central and peripheral targets in the auditory system [51].

In the SGNs, I_Na_ has a fundamental role in the spike generation [52]. Previous works have shown that DA decreases spike frequency of afferent neurons only in cases in which they are overstimulated by glutamate [22]. This is analogous with what we found. Because in isolated SGNs dopamine exerted a clear cut inhibitory effect only in those cells that were subjected to a continuous activation by sinusoidal stimuli. In cells with very low or no activity the mild effects seen in AP waveform parameters should be due to the fact that I_Na_ available for an AP generation is exceeding causing that AP generation has a large security factor. Thus, modulatory changes of the I_Na_ could not block action potential generation but modulate the discharge rate. Although DA uptake block has been shown to reduce spontaneous activity and sound-evoked compound action potential of the auditory nerve in a dose-dependent manner [53]. We found that the DA receptor activation may produce a fine adjustment of afferent neuron gain, without producing a complete block of the afferent input, thus constituting a functionally secure and efficient mechanism for gain control of the cochlear afferent activity.

## References

[1] Pujol R. Lateral and medial efferents: a double neurochemical mechanism to protect and regulate inner and outer hair cell function in the cochlea. Br J Audiol. 1994;28: 185–191. 7735146 10.3109/03005369409086567

[2] Warr WB, Guinan JJ Jr. Efferent innervation of the organ of corti: two separate systems. Brain Res. 1979;173: 152–155. 487078 10.1016/0006-8993(79)91104-1

[3] Altschuler RA, Kachar B, Rubio JA, Parakkal MH, Fex J. Immunocytochemical localization of choline acetyltransferase-like immunoreactivity in the guinea pig cochlea. Brain Res. 1985;338: 1–11. 3896390 10.1016/0006-8993(85)90242-2

[4] Eybalin M, Pujol R. Immunofluorescence with Met-enkephalin and Leu-enkephalin antibodies in the guinea pig cochlea. Hear Res. 1984;13: 135–140. 6370942 10.1016/0378-5955(84)90104-7

[5] Lu SM, Schweitzer L, Cant NB, Dawbarn D. Immunoreactivity to calcitonin gene-related peptide in the superior olivary complex and cochlea of cat and rat. Hear Res. 1987;31: 137–46. 3502259 10.1016/0378-5955(87)90119-5

[6] Eybalin M, Parnaud C, Geffard M, Pujol R. Immunoelectron microscopy identifies several types of GABA-containing efferent synapses in the guinea-pig organ of Corti. Neuroscience. 1988;24: 29–38. 3285238 10.1016/0306-4522(88)90308-9

[7] Drescher MJ, Drescher DG, Khan KM, Hatfield JS, Ramakrishnan NA, Abu-Hamdan MD, et al Pituitary adenylyl cyclase-activating polypeptide (PACAP) and its receptor (PAC1-R) are positioned to modulate afferent signaling in the cochlea. Neuroscience. 2006;142: 139–164. 16876955 10.1016/j.neuroscience.2006.05.065

[8] Altschuler RA, Hoffman DW, Wenthold RJ. Neurotransmitters of the cochlea and cochlear nucleus: immunocytochemical evidence. Am J Otolaryngol. 1986;7: 100–106. 2870655 10.1016/s0196-0709(86)80038-2

[9] Guinan JJ Jr. Cochlear efferent innervation and function. Curr Opin Otolaryngol Head Neck Surg. 2010;18: 447–453. 10.1097/MOO.0b013e32833e05d6 20717032 PMC3075443

[10] Sliwinska-Kowalska M, Parakkal M, Schneider ME, Fex J. CGRP-like immunoreactivity in the guinea pig organ of Corti: a light and electron microscopy study. Hear Res. 1989;42: 83–95. 2584160 10.1016/0378-5955(89)90119-6

[11] Bartolomé MV, Gil-Loyzaga P. Serotonergic innervation of the inner ear: is it involved in the general physiological control of the auditory receptor?. Int Tinnitus J. 2005;11: 119–125. 16639911

[12] Karadaghy AA, Lasak JM, Chomchai JS, Khan KM, Drescher MJ, Drescher DG. Quantitative analysis of DA receptor messages in the mouse cochlea. Brain Res. 1997;44: 151–156. 10.1016/s0169-328x(96)00261-6 9030711

[13] Inoue T, Matsubara A, Maruya S, Yamamoto Y, Namba A, Sasaki A, et al Localization of dopamine receptor subtypes in the rat spiral ganglion. Neurosci Lett. 2006;399: 226–229. 16490310 10.1016/j.neulet.2006.01.063

[14] Maison SF, Liu XP, Eatock RA, Sibley DR, Grandy DK, Liberman MC. Dopaminergic signaling in the cochlea: receptor expression patterns and deletion phenotypes. J Neurosci. 2012;32: 344–355. 10.1523/JNEUROSCI.4720-11.2012 22219295 PMC3313790

[15] Missale C, Nash SR, Robinson SW, Jaber M, Garon MG. Dopamine receptors: from structure to function. Physiol Rev. 1998;78: 189–225. 9457173 10.1152/physrev.1998.78.1.189

[16] Niu X, Bogdanovic N, Canlon B. The distribution and the modulation of tyrosine hydroxylase immunoreactivity in the lateral olivocochlear system of the guinea-pig. Neuroscience. 2004;125: 725–733. 15099686 10.1016/j.neuroscience.2004.02.023

[17] Gil-Loyzaga P, Parés-Herbute N. HPLC detection of dopamine and noradrenaline in the cochlea of adult and developing rats. Dev Brain Res. 1989;48: 157–160. 2752573 10.1016/0165-3806(89)90100-4

[18] Niu X, Tahera Y, Canlon B. Environmental enrichment to sound activates dopaminergic pathways in the auditory system. Physiol Behav. 2007;92: 34–39. 17631367 10.1016/j.physbeh.2007.05.020

[19] Ruel J, Nouvian R, Gervais d’Aldin C, Pujol R, Eybalin M, Puel JL. Dopamine inhibition of auditory nerve activity in the adult mammalian cochlea. Eur J Neurosci. 2001;14: 977–986. 11595036 10.1046/j.0953-816x.2001.01721.x

[20] Garrett AR, Robertson D, Sellick PM, Mulders WH. The actions of dopamine receptors in the guinea pig cochlea. Audiol Neurootol. 2010;16: 145–157. 10.1159/000316674 20668375

[21] Sun W, Salvi RJ. Dopamine modulates sodium currents in cochlear spiral ganglion neurons. Neuroreport. 2001;26: 803–807. 10.1097/00001756-200103260-00037 11277587

[22] Oestreicher E, Arnold W, Ehrenberger K, Felix D. Dopamine regulates the glutamatergic inner hair cell activity in guinea pigs. Hear Res. 1997;107: 46–52. 9165346 10.1016/s0378-5955(97)00023-3

[23] Niu X, Canlon B. The signal transduction pathway for the dopamine D1 receptor in the guinea-pig cochlea. Neuroscience. 2006;137: 981–990. 16330149 10.1016/j.neuroscience.2005.10.044

[24] Limón A, Pérez C, Vega R, Soto E. Ca^2+^-activated K^+^ current density is correlated with soma size in rat vestibular-afferent neurons in culture. J Neurophysiol. 2005;94: 3751–3761. 16107534 10.1152/jn.00177.2005

[25] Bean BP. The action potential in mammalian central neurons. Nat Rev Neurosci. 2007;8: 451–465. 17514198 10.1038/nrn2148

[26] Sunahara RK, Guan HC, O'Dowd BF, Seeman P, Laurier LG, Ng G, et al Cloning of the gene for a human dopamine D5 receptor with higher affinity for dopamine than D1. Nature. 1991;350: 614–619 1826762 10.1038/350614a0

[27] Spoendlin H. Anatomy of cochlear innervation. Am J Otolaryngol. 1985;6: 453–467. 3909832 10.1016/s0196-0709(85)80026-0

[28] Szabó ZS, Harasztosi CS, Sziclai I, Szucs G, Rusznak Z. Ionic currents determining the membrane characteristics of type I spiral ganglion neurons of the guinea pigs. J Neurosci. 2002;16: 1887–1895. 10.1046/j.1460-9568.2002.02258.x 12453052

[29] Darrow KN, Simons EJ, Dodds L, Liberman MC. Dopaminergic innervations of the mouse inner ear: evidence for a separate cytochemical group of cochlear efferent fibers. J Comp Neurol. 2006;498: 403–414. 16871528 10.1002/cne.21050 PMC1805779

[30] Arrang JM, Morisset S, Gbahou F. Constitutive activity of the histamine H3 receptor. Trends Pharmacol Sci. 2007;28: 350–357. 17573125 10.1016/j.tips.2007.05.002

[31] Kozell LB, Neve KA. Constitutive activity of a chimeric D2/D1 dopamine receptor. Mol Pharmacol. 1997;52: 1137–1149. 9396784 10.1124/mol.52.6.1137

[32] Kuroiwa M, Bateup HS, Shuto T, Higashi H, Tanaka M, Nishi A. Regulation of DARPP-32 phosphorylation by three distinct dopamine D1-like receptor signaling pathways in the neostriatum. J Neurochem. 2008;107: 1014–1026. 10.1111/j.1471-4159.2008.05702.x 18823371

[33] Carr DB, Day M, Cantrell AR, Held J, Scheuer T, Catterall WA, et al Transmitter modulation of slow, activity-dependent alterations in sodium channel availability endows neurons with a novel form of cellular plasticity. Neuron. 2003;39: 793–806. 12948446 10.1016/s0896-6273(03)00531-2

[34] Cantrell AR, Scheuer T, Catterall WA. Voltage-dependent neuromodulation of Na^+^ channels by D1-like dopamine receptors in rat hippocampal neurons. J Neurosci. 1999;19: 5301–5310. 10377341 10.1523/JNEUROSCI.19-13-05301.1999 PMC6782346

[35] Lee FJ, Xue S, Pei L, Vukusic B, Chéry N, Wang Y, et al Dual regulation of NMDA receptor functions by direct protein-protein interactions with the dopamine D1 receptor. Cell. 2002;111: 219–230. 12408866 10.1016/s0092-8674(02)00962-5

[36] Pei L, Lee FJ, Moszczynska A, Vukusic B, Liu F. Regulation of dopamine D1 receptor function by physical interaction with the NMDA receptors. J Neurosci. 2004;24: 1149–1158. 14762133 10.1523/JNEUROSCI.3922-03.2004 PMC6793575

[37] Liu F, Wan Q, Pristupa ZB, Yu XM, Wang YT, Niznik HB. Direct protein-protein coupling enables cross-talk between dopamine D5 and gamma-aminobutyric acid A receptors. Nature. 2000;403: 274–280. 10659839 10.1038/35002014

[38] Franco R, Lluis C, Canela EI, Mallol J, Agnati L, Casadó V, et al Receptor-receptor interactions involving adenosine A1 or dopamine D1 receptors and accessory proteins. J Neural Transm. 2007;114: 93–104. 17024327 10.1007/s00702-006-0566-7

[39] Cabello N, Gandía J, Bertarelli DC, Watanabe M, Lluís C, Franco R, et al Metabotropic glutamate type 5, dopamine D2 and adenosine A2a receptors form higher-order oligomers in living cells. J Neurochem. 2009;109: 1497–1507. 10.1111/j.1471-4159.2009.06078.x 19344374 PMC3925975

[40] Ferrada C, Moreno E, Casadó V, Bongers G, Cortés A, Mallol J, et al Marked changes in signal transduction upon heteromerization of dopamine D1 and histamine H3 receptors. Br J Pharmacol. 2009;157: 64–75. 10.1111/j.1476-5381.2009.00152.x 19413572 PMC2697789

[41] Maurice N, Mercer J, Chan CS, Hernandez-Lopez S, Held J, Tkatch T, et al D2 dopamine receptor-mediated modulation of voltage-dependent Na^+^ channels reduces autonomous activity in striatal cholinergic interneurons. J Neurosci. 2004;24: 10289–10301. 15548642 10.1523/JNEUROSCI.2155-04.2004 PMC6730305

[42] Lee FJ, Liu F. Direct interactions between NMDA and D1 receptors: a tale of tails. Biochem Soc Trans. 2004;32: 1032–1036. 15506956 10.1042/BST0321032

[43] Rashid AJ, So CH, Kong MM, Furtak T, El-Ghundi M, Cheng R, et al D1-D2 dopamine receptor heterooligomers with unique pharmacology are coupled to rapid activation of Gq/11 in the striatum. Proc Natl Acad Sci U S A. 2007;104: 654–659. 17194762 10.1073/pnas.0604049104 PMC1766439

[44] Verma V, Hasbi A, O'Dowd BF, George SR. Dopamine D1-D2 Receptor Heteromer-mediated Calcium Release Is Desensitized by D1 Receptor Occupancy with or without Signal Activation: Dual functional regulation by G-protein-coupled receptor kinase 2. J Biol Chem. 2010;285: 35092–35103. 10.1074/jbc.M109.088625 20807772 PMC2966123

[45] Perreault ML, Hasbi A, O'Dowd BF, George SR. The dopamine d1-d2 receptor heteromer in striatal medium spiny neurons: evidence for a third distinct neuronal pathway in Basal Ganglia. Front Neuroanat. 2011;5: 31 10.3389/fnana.2011.00031 21747759 PMC3130461

[46] Mo ZL, Davis RL. Heterogeneous voltage dependence of inward rectifier currents in spiral ganglion neurons. J Neurophysiol. 1997;78: 3019–3027. 9405521 10.1152/jn.1997.78.6.3019

[47] Adamson CL, Reid MA, Davis RL. Opposite actions of brain-derived neurotrophic factor and neurotrophin-3 on firing features and ion channel composition of murine spiral ganglion neurons. J Neurosci. 2002;22: 1385–1396. 11850465 10.1523/JNEUROSCI.22-04-01385.2002 PMC6757552

[48] d'Aldin C, Puel JL, Leducq R, Crambes O, Eybalin M, Pujol R. Effects of a dopaminergic agonist in the guinea pig cochlea. Hear Res. 1995;90: 202–211. 8974998 10.1016/0378-5955(95)00167-5

[49] Gil-Loyzaga P, Vicente-Torres MA, Fernández-Mateos P, Arce A, Esquifino A. Piribedil affects dopamine turnover in cochlea stimulated by white noise. Hear Res. 1994;79: 178–182. 7806480 10.1016/0378-5955(94)90138-4

[50] Polony G, Humli V, Andó R, Aller M, Horváth T, Harnos A, et al Protective effect of rasagiline in aminoglycoside ototoxicity. Neuroscience. 2014;265: 263–273. 10.1016/j.neuroscience.2014.01.057 24508748

[51] Sziklai I, Szilvássy J, Szilvássy Z. Tinnitus control by dopamine agonist pramipexole in presbycusis patients: A randomized, placebo-controlled, double-blind study. Laryngoscope. 2011;121: 888–893. 10.1002/lary.21461 21433025

[52] Hossain WA, Antic SD, Yang Y, Rasband MN, Morest DK. Where is the spike generator of the cochlear nerve? Voltage-gated sodium channels in the mouse cochlea. J Neurosci. 2005;25: 6857–6868. 16033895 10.1523/JNEUROSCI.0123-05.2005 PMC1378182

[53] Ruel J, Wang J, Demêmes D, Gobaille S, Puel JL, Rebillard G. Dopamine transporter is essential for the maintenance of spontaneous activity of auditory nerve neurones and their responsiveness to sound stimulation. J Neurochem. 2006;97: 190–200. 16524378 10.1111/j.1471-4159.2006.03722.x

